# Disentangling the contributions of maternal and fetal factors to estimate stillbirth risks for intrapartum adverse events in Tanzania and Uganda

**DOI:** 10.1002/ijgo.12689

**Published:** 2018-10-26

**Authors:** Atsumi Hirose, Fadhlun Alwy, Susan Atuhairwe, Jessica L. Morris, Andrea B. Pembe, Frank Kaharuza, Gaetano Marrone, Claudia Hanson

**Affiliations:** ^1^ Department of Public Health Sciences Karolinska Institutet Stockholm Sweden; ^2^ Department of Obstetrics and Gynaecology Muhimbili University of Health and Allied Sciences Dar es Salaam Tanzania; ^3^ Association of Gynaecologists and Obstetricians of Tanzania Dar es Salaam Tanzania; ^4^ Association of Obstetricians and Gynaecologists of Uganda Kampala Uganda; ^5^ Mulago National Referral Hospital Kampala Uganda; ^6^ International Federation of Gynecology and Obstetrics London UK; ^7^ Makerere University School of Public Health Kampala Uganda; ^8^ Department of Disease Control London School of Hygiene and Tropical Medicine London UK

**Keywords:** Epidemiology, Fetal deaths, Near‐miss, Organ dysfunction, Severe maternal morbidity, Stillbirths, Tanzania, Uganda

## Abstract

**Objective:**

To estimate the stillbirth risk associated with intrapartum adverse events, controlling for fetal and maternal factors.

**Methods:**

The present study was an analysis of cross‐sectional patient‐record and facility‐file data from women with viable fetuses who experienced obstetric adverse events at 23 hospitals and 38 health centers in Tanzania (between December 2015 and October 2016), and 22 hospitals, 16 level‐4 health centers, and five level‐3 health centers in Uganda (between May 2016 and September 2017). Adverse events were categorized in three severity groups (postpartum, intrapartum non‐near‐miss, and intrapartum near‐miss) to calculate stillbirth rates and adjusted prevalence ratios.

**Results:**

Data from 3816 women in Tanzania and 8305 in Uganda were included. Compared with postpartum adverse events, intrapartum near‐miss was associated with a 3.73‐ and 4.55‐fold higher prevalence of stillbirth in Uganda and Tanzania, respectively. Most women who experienced near‐miss had organ dysfunction on arrival or developed it soon after. The risk of stillbirth was higher among preterm deliveries compared with term deliveries, and was 42% and 59% lower in Tanzania and Uganda, respectively, for cesarean deliveries compared with vaginal deliveries after intrapartum non‐near‐miss adverse events.

**Conclusion:**

Stillbirth risk increased with severity of complications and was higher among premature deliveries. Survival was higher for cesarean deliveries in intrapartum non‐near‐miss complications, identifying the opportunity to prevent deterioration by timely actions.

## Abstract 2

Risk of stillbirth increased with severity of maternal complications. Prompt action to prevent the development of maternal near‐miss events has the potential to prevent stillbirth.

## 1 Introduction

Perinatal mortality and morbidity are intimately linked to maternal mortality and morbidity. In recent years, the severe end of maternal morbidity, often referred to as maternal near‐miss, has received recognition as an important indicator to assess healthcare service and program performance.^1^ Since standardization of the maternal morbidity and near‐miss concepts,^1^, ^2^, ^3^ facility‐based incidents of maternal morbidity have been reported from high‐, middle‐, and low‐income countries. Partly linked to measurement challenges in definition and data capturing,^4^ however, there is a paucity of studies analyzing stillbirth or quantifying the risk among near‐miss women; this is despite the general understanding that delivery outcomes are particularly sensitive to the quality of intrapartum care and management of complications.

Stillbirth is associated with obstetric complications such as prepartum hemorrhage, ruptured uterus, pre‐eclampsia, eclampsia, maternal anemia, and infection.^5^, ^6^ Fetal conditions associated with stillbirth include post‐term pregnancy, small for gestational age, low birthweight, prematurity, and multiple gestations.^6^, ^7^, ^8^ Maternal complications explain many severe fetal morbidities and mortalities during delivery, and thus appropriate and timely management of these complications has the potential to avoid many adverse fetal outcomes.^9^ The large disparity in stillbirth rates seen in maternal near‐miss cases across different countries, from 3.8% in Finland to as high as 46% in low‐ to middle‐income countries,^10^, ^11^, ^12^, ^13^, ^14^ is probably attributable to substandard management of complications. However, measurement challenges prevail in low‐resource settings,^15^ restricting a direct comparisons of rates across different settings.

The aim of the present study was to clarify the role of the severity of maternal complications or maternal near‐miss in stillbirth, after taking into account associations between maternal (obstetric and reproductive) factors and fetal conditions in datasets from two low‐income countries, Tanzania and Uganda. Maternal and fetal factors could be causal factors or consequences of maternal near‐miss events; disentangling the relationships will enable the risk of stillbirth to be estimated in maternal near‐miss events.

## 2 Materials and Methods

The present study used cross‐sectional data collected as part of a trial evaluating the effects of 1‐day competency‐based “Helping Mothers Survive Bleeding After Birth” (HMS BAB) training to reduce postpartum hemorrhage (PPH)‐related morbidity and mortality in Tanzania between December 1, 2015, and October 31, 2016, and in Uganda between May 1, 2016, and September 30, 2017. Ethical approval was obtained from the institutional review board of Muhimbili University of Health and Allied Sciences and the Commission of Science and Technology, Dar es Salaam, Tanzania, and from the Makerere University School of Medicine Research and Ethics Committee and the Uganda National Council for Science and Technology, Kampala, Uganda. Requiring informed consent was waived by the boards that approved the study because the data were anonymized, and identification of individuals not possible.

Details of the HMS BAB trial are described elsewhere.^16^ In Tanzania, 23 hospitals and 38 health centers in 20 districts were included. All were government‐owned facilities except for six mission hospitals or clinics. In Uganda, 22 hospitals, 16 level IV health centers, and five high‐volume level III health centers offering some emergency obstetric care services were included. All were government‐owned except for eight faith‐based facilities and one NGO facility.

The WHO near‐miss tool^2^ was adapted for the study (File S1). Maternity staff received a short training, after which they reviewed the prenatal, delivery, and postnatal registries, and patient case notes on a daily basis to identify women with complications. Data were abstracted by using the tool to capture information on obstetric complications (PPH, severe pre‐eclampsia, eclampsia, sepsis/severe infection, ruptured uterus, severe complications of induced and spontaneous abortions, and prepartum hemorrhage), critical interventions, organ dysfunctions, maternal outcome, mode of delivery or end of pregnancy, and vital status of the neonate at delivery. The tool was similar in both countries, but minor adaptations were made to address country differences in data collection and practices. For example, the timing of a stillbirth (before or during delivery) was estimated by appearance of the skin in Uganda (fresh or macerated) but not in Tanzania. Stillbirths were not weighed in Tanzania and hence birthweight data were not collected.

Women were included in the study if they delivered a potentially viable fetus (≥1000 g or, if birthweight was unknown, ≥28 weeks of pregnancy) and experienced PPH, prepartum hemorrhage, eclampsia/pre‐eclampsia, sepsis, or ruptured uterus.

To classify the degree of severity and its potential effect on stillbirth, women were categorized on the basis of complications into three mutually exclusive risk groups (Table S1): a low‐risk group, including those experiencing “postpartum complications” only (in the present study, PPH only); a medium‐risk group including those experiencing prepartum or intrapartum complications without organ dysfunction or those without management‐based criteria indicating severity (blood transfusion or hysterectomy) (collectively termed “intrapartum non‐near‐miss complications”); and a high‐risk group including those experiencing prepartum or intrapartum complications and any organ dysfunction and/or management‐based criteria indicating severity (termed “intrapartum near‐miss complications”). As suggested by Nelissen et al.,^17^ the threshold of blood transfusion was lowered from 5 units to 2 units to define coagulation/hematologic dysfunction.^17^

Analysis followed the conceptual framework (Fig. S1), which was based on previous studies.^4^, ^5^ Underlying causes contributing to stillbirth, factors associated with (but not directly contributing to) stillbirth, and factors on the causal pathway to stillbirth were considered separately in the analysis. Factors associated with stillbirth, in particular maternal factors (age and parity), were considered potential confounders because they might also relate to maternal complications. Place and mode of delivery, and a fetal factor (gestational age) were on the causal pathway between complications and stillbirth, and therefore stratified analyses were conducted. Immediate causes of stillbirth (e.g., asphyxia or infection) were not measured and thus not included in analysis.

All statistical analyses were conducted with Stata version 13 (StataCorp, College Station, TX, USA) using Stata survey commands to take the clustering within facilities into account. First, factors were compared among the three risk groups. The stillbirth rate was calculated per 1000 complicated deliveries, defined as the number of stillbirths divided by the number of live deliveries and stillbirths exposed to obstetric complications, and the rates related to risk groups and key factors were estimated. Multivariable Poisson regression models were used to estimate the adjusted prevalence ratio (aPR) of stillbirth in risk groups for ease of interpretation.^18^

Stratified analysis was used to explore the mediating effects on stillbirth of factors on the causal pathways. Outcomes were imputed by using multiple imputation techniques of 20 data sets and the estimates were combined by using Rubin rules.^19^ A sensitivity analysis was used to compare the results between observed data and imputed data (Fig. S2 and Tables [Link], [Link], [Link]). *P*<0.05 was considered to be significant.

## 3 Results

**Figure 1:**
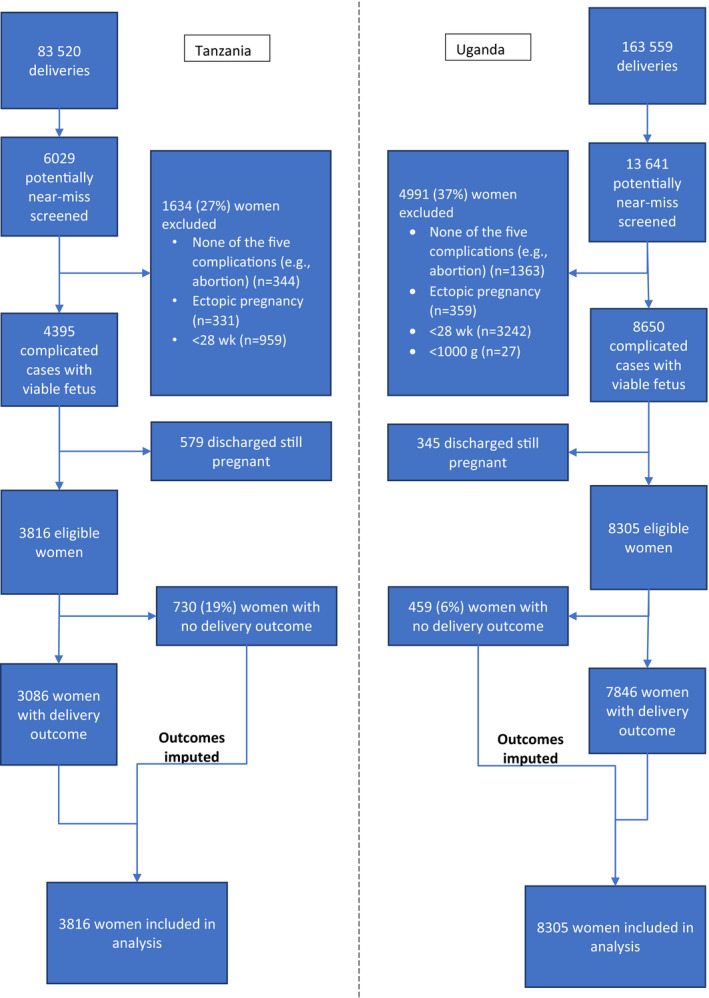
Flow chart of the patient inclusion in Tanzania and Uganda.

During the study period, 83 520 and 163 559 deliveries were reported. Because the data were obtained from routine recording systems, delivery outcomes were missing for 730 and 459 deliveries in Tanzania and Uganda, respectively. There was no significant difference in maternal, delivery, and fetal factors between those with delivery outcomes and those without in Tanzania, except that mode of delivery was more likely to be unknown for women missing an outcome, and the majority of those without outcomes had intrapartum non‐near‐miss complications (Table S2). In Uganda, the majority of missing outcomes were from data collected in health centers and for women who had intrapartum near‐miss complications. The mode of delivery for most women with missing outcomes was unknown (Table 2). In total, 3816 (654 high‐risk, 1416 medium‐risk, and 1746 low‐risk) and 8305 (2374 high‐risk, 1644 medium‐risk, and 4287 low‐risk) deliveries with obstetric complications in Tanzania and Uganda, respectively, were included in the study (Fig. 1).

The most common complication of delivery was PPH, followed by hypertensive disorders (1001 [26.2%] and 1627 [19.6%] in Tanzania and Uganda, respectively) (Table 1). The medium‐risk group (intrapartum non‐near‐miss complications) consisted largely of deliveries complicated by hypertensive disorders (808 [57%] and 848 [52%] in Tanzania and Uganda, respectively), whereas the high‐risk group (intrapartum near‐miss complications) was more equally proportioned among hypertensive disorders, prepartum hemorrhage, and infection: 22.5% (147/654) and 22.8% (540/2374) involved rupture of the uterus in Tanzania and Uganda, respectively. Women in the intrapartum near‐miss group were slightly older (median age, 26 and 25 years in Tanzania and Uganda, respectively), whereas those in the intrapartum non‐near‐miss group were younger (23 and 24 years, respectively). As compared with the postpartum complications group, more women in the two intrapartum groups delivered by cesarean. The proportion of preterm deliveries was very high: 31.4% (1197/3816) and 18.7% (1555/8305) of all complicated deliveries, and 37.3% (244/654) and 27.6% (655/2374) of deliveries in the intrapartum near‐miss group in Tanzania and Uganda, respectively (Table 1). In Tanzania, the most common organ dysfunction was coagulation/hematologic dysfunction (373/654 [57.0%]), followed by cardiovascular dysfunction. In Uganda, cardiovascular dysfunction was most common (1411/2374 [59.4%]), followed by coagulation dysfunction. Most women who had organ dysfunctions had them on arrival at the health facility or developed them within 12 hours of arrival (Fig. S3).

**Table 1:** Underlying causes, and maternal and fetal risk factors by severity of obstetric complications in Tanzania and Uganda.a

Causes and risk factors	Tanzania					Uganda				
	Total (n=3816)	Postpartum complication (low risk) (n=1746)	Intrapartum non‐near‐miss (medium risk) (n=1416)	Intrapartum near‐miss (high risk)^a^ (n=654)	*P* value^b^	Total (n=8305)	Postpartum complication (low risk) (n=4287)	Intrapartum non‐near‐miss (medium risk) (n=1644)	Intrapartum near‐miss (high risk)^a^ (n=2374)	*P* value^b^
Underlying cause										
Maternal complications^c^										
Postpartum hemorrhage	1889 (49.5)	1746 (100.0)	42 (3.0)	101 (15.4)	<0.001	5076 (61.1)	4287 (100.0)	185 (11.3)	604 (25.4)	<0.001
Hypertensive disorders	1001 (26.2)	0	808 (57.1)	193 (29.5)	<0.001	1627 (19.6)	0	848 (51.6)	779 (32.8)	<0.001
Prepartum hemorrhage	495 (13.0)	0	300 (21.2)	195 (29.8)	<0.001	1147 (13.8)	0	521 (31.7)	626 (26.4)	<0.001
Infection	475 (12.5)	0	312 (22.0)	196 (24.9)	<0.001	1059 (12.8)	0	236 (14.4)	823 (34.7)	<0.001
Uterine rupture	167 (4.4)	0	20 (1.4)	147 (22.5)	<0.001	620 (7.5)	0	80 (4.9)	540 (22.8)	<0.001
Maternal risk factors										
Age, y					0.008					0.041
<20	890 (23.3)	393 (22.5)	368 (26.0)	129 (19.7)		1600 (19.3)	859 (20.0)	337 (20.5)	404 (17.0)	
20–24	976 (25.6)	427 (24.5)	392 (27.7)	157 (24.0)		2599 (31.3)	1381 (32.2)	495 (30.1)	723 (30.5)	
25–29	704 (18.5)	316 (18.1)	392 (18.3)	129 (19.7)		1855 (22.3)	950 (22.2)	374 (22.8)	531 (22.4)	
30–34	587 (15.4)	289 (16.6)	197 (13.9)	101 (15.4)		1245 (15.0)	609 (14.2)	240 (14.6)	396 (16.7)	
35–39	433 (11.4)	205 (11.7)	136 (9.6)	92 (14.1)		783 (9.4)	383 (8.9)	158 (9.6)	242 (10.2)	
≥40	226 (5.9)	116 (6.6)	64 (4.5)	46 (7.0)		223 (2.7)	105 (2.4)	40 (2.4)	78 (3.3)	
Median age, y	25	25	23	26	<0.001^d^	24	24	24	25	<0.001^d^
Parity					0.005					0.229
0	1300 (34.4)	551 (31.6)	561 (39.6)	188 (28.8)		1656 (19.9)	795 (18.5)	361 (22.0)	500 (21.1)	
1	719 (18.8)	315 (18.1)	262 (18.5)	142 (21.7)		1869 (22.5)	952 (22.2)	419 (25.5)	498 (21.0)	
2	529 (13.9)	229 (13.1)	201 (14.2)	98 (15.0)		1311 (15.8)	706 (16.5)	239 (14.5)	366 (15.4)	
≥3	1268 (33.2)	651 (37.3)	392 (27.7)	226 (34.6)		3469 (41.8)	1834 (42.8)	625 (38.0)	1010 (42.5)	
Delivery factors										
Delivery mode					<0.001					<0.001
Vaginal	2433 (63.8)	1506 (86.3)	729 (51.5)	198 (30.3)		3967 (47.8)	2987 (69.7)	457 (27.8)	523 (22.0)	
Cesarean/surgery	1274 (33.4)	234 (13.4)	602 (42.5)	438 (67.0)		3959 (47.7)	1290 (30.1)	1068 (65.0)	1601 (67.4)	
Unknown	109 (2.9)	6 (0.3)	85 (6.0)	18 (2.8)		379 (4.6)	10 (0.2)	119 (7.2)	250 (10.5)	
Delivery place					0.004					<0.001
Study hospital	2666 (69.9)	1093 (62.6)	1076 (76.0)	497 (76.0)		5516 (66.4)	2391 (55.8)	1325 (80.6)	1800 (75.8)	
Study health center	604 (15.8)	414 (23.7)	107 (7.6)	83 (12.7)		1830 (22.0)	1227 (28.6)	227 (13.8)	376 (15.8)	
Other facility and referred	113 (3.0)	76 (4.4)	14 (1.0)	23 (3.5)		558 (6.7)	412 (9.6)	40 (2.4)	106 (4.5)	
Other	433 (11.4)	163 (9.3)	219 (15.5)	51 (7.8)		401 (4.8)	257 (6.0)	52 (3.2)	92 (3.9)	
Fetal factors										
GA					0.004					<0.001
Preterm (<37 wk)	1197 (31.4)	416 (23.8)	537 (37.9)	244 (37.3)		1555 (18.7)	471 (11.0)	429 (26.1)	655 (27.6)	
Term	2483 (65.1)	1268 (72.4)	845 (59.7)	370 (56.6)		5665 (68.2)	3212 (74.9)	1060 (64.5)	1393 (58.7)	
Post term	31 (0.8)	19 (1.1)	8 (0.6)	4 (0.6)		42 (0.5)	26 (0.6)	3 (0.2)	13 (0.5)	
Missing	105 (2.8)	43 (2.5)	26 (1.8)	36 (5.5)		1043 (12.6)	578 (13.5)	152 (9.2)	313 (13.2)	
Birthweight, kg^e^										<0.001
<2.5						996 (12.0)	302 (7.0)	298 (18.1)	396 (16.7)	
2.5–4.0						5239 (63.1)	3117 (72.7)	934 (56.8)	1188 (50.0)	
≥4.0						474 (5.7)	311 (7.3)	69 (4.2)	94 (4.0)	
Missing						1596 (19.2)	557 (13.0)	343 (20.9)	696 (29.3)	

Abbreviation: GA, gestational age.

Data are given as number (percentage) unless indicated otherwise.

a Near‐miss includes women having intrapartum complications with organ dysfunctions.

b χ^2^ test unless stated otherwise.

c Maternal complications are described using five independent variables. Because some women had more than one complication, it is not possible to create five mutually exclusive five.

d Kruskal‐Wallis test.

e Birthweight not measured in Tanzania.

The overall stillbirth rate was 133 per 1000 complicated deliveries (95% confidence interval [CI] 101–164) in Tanzania and 151 (95% CI 124–177) in Uganda. Of all stillbirths, 74% were estimated to have occurred during delivery in Uganda (data not shown). The crude stillbirth rate was significantly higher among women who delivered by cesarean than among those who had a vaginal delivery (188 vs 95 [*P*<0.001] and 185 vs 107 [*P*<0.001] per 1000 complicated deliveries in Tanzania and Uganda, respectively) and among hospital‐based deliveries than among lower‐level facilities (145 vs 88 [*P*=0.002] and 168 vs 98 [*P*<0.001] per 1000 complicated deliveries, respectively). More than one‐fifth of preterm deliveries in both countries and of low birthweight deliveries in Uganda were stillbirths (Table 2).

**Table 2:** Frequency of stillbirths during complicated deliveries by maternal, delivery, and fetal factors

Factor	Tanzania				Uganda			
	No. of deliveries	% of live deliveries^a^	% of stillbirths^a^	Stillbirth rate per 1000 complicated deliveries (95% CI)	No. of deliveries	% of live deliveries^a^	% of stillbirths^a^	Stillbirth rate per 1000 complicated deliveries (95% CI)
Overall	3816			132.6 (101.2–163.9)	8305			150.6 (124.4–176.8)
Maternal factors								
Age, y								
<20	890	24.4	16.2	92.2 (64.4–120.1)	1600	20.2	13.8	107.9 (84.9–130.8)
20–24	976	25.7	24.5	126.9 (92.7–161.1)	2599	31.7	28.8	138.6 (112.7–164.6)
25–29	704	18.2	20.3	146.2 (103.3–189.0)	1855	22.5	21.6	145.6 (111.7–179.5)
30–34	587	15.1	17.0	146.7 (95.3–198.0)	1245	14.1	19.9	199.4 (158.0–240.9)
35–39	433	10.9	14.1	164.3 (112.4–216.3)	783	8.9	12.4	198.0 (157.3–238.8)
≥40	226	5.6	7.9	176.3 (101.4–251.3)	223	2.5	3.5	198.4 (139.2–257.7)
Parity								
0	1299	35.1	27.2	106.1 (79.6–132.6)	1656	21.0	14.0	105.5 (83.2–127.9)
1	718	18.8	19.0	134.1 (93.5–174.6)	1869	23.3	18.0	120.4 (88.7–152.2)
2	528	13.6	15.8	151.0 (94.5–207.4)	1311	15.9	15.4	146.9 (116.5–177.2)
≥3	1267	32.5	38.0	151.5 (104.8–198.2)	3469	39.8	52.6	189.7 (157.3–222.1)
Delivery factors								
Mode of delivery								
Vaginal	2433	66.5	45.9	95.4 (73.5–117.3)	3967	50.2	34.1	107.4 (90.9–123.9)
Cesarean/surgery	1274	31.2	47.4	188.3 (133.9–242.7)	3959	45.7	58.7	185.3 (139.9–230.7)
Unknown	109	2.3	6.7	311.5 (118.3–504.6)	379	4.1	7.2	239.6 (100.4–378.7)
Place of delivery								
Study hospital	2666	68.8	76.6	145.4 (104.8–186.0)	5516	65.0	74.2	168.2 (132.3–204.1)
Study health center	604	16.6	10.5	87.8 (46.2–129.4)	1830	23.4	14.4	98.3 (68.1–128.4)
Other facility and referred	113	3.0	2.8	126.1 (65.4–186.8)	558	6.5	7.8	173.7 (133.4–214.1)
Other	433	11.5	10.1	117.8 (49.8–185.8)	401	5.0	3.7	115.0 (80.0–149.9)
Fetal factors								
GA								
Preterm (<37 wk)	1197	28.8	48.3	204.3 (163.1–245.6)	1555	16.6	30.8	248.0 (208.5–287.5)
Term	2483	67.9	46.3	94.3 (67.7–121.0)	5665	70.6	55.0	121.4 (91.9–151.0)
Post term	31	0.9	0.5	77.4 (−12.9 to 167.8)	42	0.6	0.2	47.6 (−37.7 to 132.9)
Missing	105	2.4	4.9	235.7 (117.7–353.7)	1043	12.3	14.0	167.8 (133.5–202.2)
Birthweight, kg^b^								
<2.5					996	11.2	16.7	209.6 (177.5–241.8)
2.5–4.0					5239	67.1	40.3	96.2 (71.5–120.8)
≥4.0					474	5.8	5.3	139.2 (90.4–188.1)
Missing					1596	15.9	37.7	295.7 (247.1–344.4)

Abbreviation: CI, confidence interval; GA, gestational age.

a Live delivery and stillbirth rates calculated using data imputation; consequently, absolute numbers are not available.

b Birthweight not measured in Tanzania.

**Figure 2:**
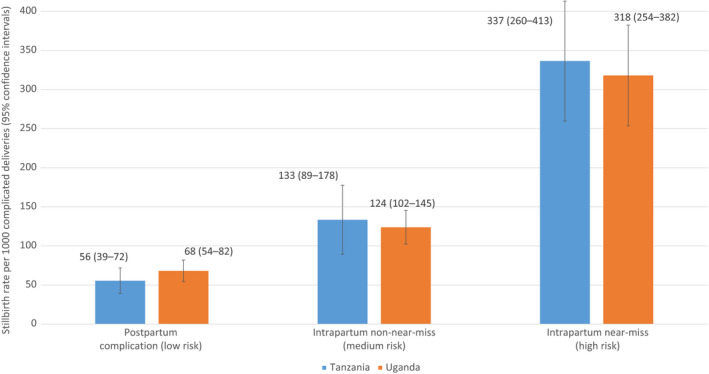
Stillbirth rates per 1000 complicated deliveries stratified by risk group in Tanzania and Uganda.

In both countries, the stillbirth rate was significantly higher in the intrapartum near‐miss group than in the intrapartum non‐near‐miss group (337 vs 133 [*P*<0.001] and 318 vs 124 [*P*<0.001] per 1000 complicated deliveries in Tanzania and Uganda, respectively), which was in turn significantly higher than in the postpartum complication group (*P*<0.001 in both Tanzania and Uganda) (Fig. 2). Between the high‐risk intrapartum near‐miss group and the low‐risk postpartum complication group, there was an approximately fourfold increase in risk of stillbirth (aPR 4.55, 95% CI 2.94–7.04 in Tanzania; and aPR 3.73, 95% CI 2.86–4.88 in Uganda) (Table S6).

Stratified analysis showed that the prevalence of stillbirth more than doubled for preterm deliveries as compared with term deliveries in the postpartum and intrapartum non‐near‐miss groups and increased by 68% in the near‐miss group, after adjustment for types of complication, maternal age, and parity in Tanzania (Table 3). The prevalence ratio of stillbirth for preterm deliveries relative to term deliveries varied between 1.27 and 3.99 across the three risk groups in Uganda.

**Table 3:** Stillbirth rates stratified by delivery mode, gestational age, and place of delivery in Tanzania and Uganda

Factor	Postpartum complication (low risk)			Intrapartum non‐near‐miss (medium risk)			Intrapartum near‐miss (high risk)^a^		
	Stillbirth rate (95%CI)	*P* value	aPR^b^	Stillbirth rate (95% CI)	*P* value	aPR^b^	Stillbirth rate (95% CI)	*P* value	aPR^b^
Tanzania									
Gestational age at delivery									
Preterm	89.7 (56.0–123.3)	0.001	2.17 (1.39–3.39)	197.3 (142.0–252.6)	<0.001	2.29 (1.69–3.12)	415.4 (327.0–503.7)	0.001	1.68 (1.24–2.26)
Term	42.7 (26.3–59.0)	Ref.	1.00	89.2 (41.1–137.4)	Ref.	1.00	283.0 (197.7–368.2)	Ref.	1.00
Delivery mode									
Cesarean	44.7 (10.4–78.9)	0.498	0.79 (0.39–1.58)	112.8 (68.3–157.3)	0.006	0.58 (0.39–0.85)	368.8 (277.3–460.4)	0.245	0.79 (0.53–1.17)
Vaginal	55.6 (38.7–72.8)	Ref.	1.00	137.4 (84.5–190.2)	Ref.	1.00	242.4 (155.1–329.7)	Ref.	1.00
Delivery place									
Hospital	50.6 (33.0–68.2)	Ref.	1.00	138.6 (94.3–182.9)	Ref.	1.00	368.7 (279.9–457.5)	Ref.	1.00
Health center	37.2 (12.5–61.9)	0.201	0.66 (0.34–1.25)	146.3 (59.9–232.6)	0.937	0.98 (0.54–1.76)	265.1 (62.6–467.5)	0.411	0.82 (0.51–1.32)
Uganda									
Gestational age at delivery									
Preterm	183.3 (122.8–243.9)	<0.001	3.99 (3.05–5.22)	211.5 (168.0–255.1)	<0.001	2.36 (1.70–3.27)	318.3 (242.8–393.9)	0.012	1.27 (1.05–1.53)
Term	45.4 (32.7–58.1)	Ref.	1.00	92.2 (65.9–118.6)	Ref.	1.00	319.1 (244.3–393.9)	Ref.	1.00
Delivery mode									
Cesarean	70.3 (41.4–99.3)	0.754	1.04 (0.81–1.34)	101.0 (78.1–123.9)	<0.001	0.41 (0.30–0.56)	334.2 (253.2–415.2)	0.005	0.74 (0.60–0.92)
Vaginal	67.4 (54.5–80.2)	Ref.	1.00	172.4 (140.9–203.9)	Ref.	1.00	279.3 (230.0–328.5)	Ref.	1.00
Delivery place									
Hospital	72.3 (51.9–92.6)	Ref.	1.00	118.3 (96.6–140.1)	Ref.	1.00	332.3 (256.4–408.2)	Ref.	1.00
Health center	38.7 (26.4–51.0)	<0.001	0.53 (0.38–0.73)	150.9 (75.3–226.4)	0.856	0.96 (0.60–1.52)	260.8 (165.2–356.4)	0.992	1.00 (0.74–1.35)

Abbreviation: aPR, adjusted prevalence ratio; CI, confidence interval.

a Near‐miss includes women with intrapartum complications and organ dysfunction.

b Adjusted for maternal age, parity, and complication types.

After adjustment, a 42% (aPR 0.58, 95% CI 0.39–0.85) and a 59% (aPR 0.41, 95% CI 0.30–0.56) reduced risk of stillbirth in cesarean as compared with vaginal deliveries was observed in the intrapartum non‐near‐miss group in Tanzania and Uganda, respectively. In Uganda, a 26% (aPR 0.74, 95% CI 0.60–0.92) reduced risk of stillbirth in cesarean as compared with vaginal deliveries was observed in the intrapartum near‐miss group after adjustment. No significant difference in stillbirth rates were observed between hospital‐based and health‐center‐based deliveries among intrapartum complication groups.

## 4 Discussion

The present large study, including 3816 and 8305 complicated deliveries in Tanzania and Uganda, respectively, found that there was a 4.55‐ and 3.73‐fold higher risk of stillbirth risk when intrapartum complications developed into a near‐miss situation. The risk of stillbirth was significantly lower for term than for preterm deliveries, and for cesarean than for vaginal deliveries, particularly when the complication did not develop into a near‐miss situation.

The association between maternal near‐miss and stillbirth has rarely been quantified in low‐ and middle‐income countries. One multi‐country study from Latin America reported an almost fourfold higher risk of stillbirth for women experiencing any near‐miss complication, as compared with non‐near‐miss (including uncomplicated) deliveries, similar to the present finding. However, the Latin American study reported much lower stillbirth rates for maternal near‐miss deliveries (37 per 1000 near‐miss deliveries) as compared with the present estimate.^20^

The present high stillbirth rates are consistent with those of a previous Ugandan study, reporting 120 stillbirths per 1000 deliveries with severe complications in a referral hospital, but lower than those of a Nigerian study, which documented 211 stillbirths per 1000 deliveries with severe maternal morbidity in a tertiary hospital.^12^, ^21^ Furthermore, the present study supports earlier findings that preterm delivery is a risk factor for stillbirth.^22^ Considering the high prevalence of preterm deliveries among women with complications, the number of stillbirths associated with preterm delivery may be greater than suggested previously. The global prevalence of preterm delivery among the general population in low‐income countries is estimated to be 11.8%.^23^ In the present study, 37% and 28% of intrapartum near‐miss women delivered preterm in Tanzania and Uganda.

Access to timely, high‐quality intrapartum care is essential for the prevention of intrapartum stillbirth. Although the study hospitals were equipped to provide comprehensive emergency obstetric and neonatal care, the survival of neonates was poor in hospital deliveries. In maternal near‐miss events, most women had a near‐miss condition on arrival or within 12 hours of admission, suggesting that delays before or on admission contributed to the high stillbirth rate. Moreover, three‐quarters of the stillbirths in Uganda were reported to have occurred during delivery.

As expected, the postpartum complication group had a lower risk than the intrapartum groups because complications occurred after delivery. Nevertheless, the stillbirth rate was still higher than estimates among the general population,^24^ which may support the hypothesis that there is a pathological link between PPH and other obstetric complications such as undiagnosed hypertensive disorders (i.e., HELLP syndrome) and gestational diabetes, which leads to placenta disorders. In addition, in two‐thirds of the patients with reported PPH, delivery was by cesarean. Although the reason for the cesarean was not recorded, it is likely that many procedures were performed for obstetric complications, including prolonged labor or fetal distress, which might explain the high number of stillbirths.

A strength of the study is the application of the WHO maternal near‐miss tool in a large number of facilities in Uganda and Tanzania. This allowed standardization of the criteria for severity of maternal outcomes and analysis of relatively large samples from differing settings, thereby increasing the generalizability of the findings. It is also one of a few studies that have quantified stillbirth risks among patients experiencing maternal near‐miss events and highlighted the link between the two.

The study also has limitations. First, the study was based on routine data collected in several facilities. Uncertainty exists in pregnancy dating because early dating scans are not available in these settings. There is also uncertainty in the timing of stillbirth in the Ugandan sample. It would have been preferable to use the presence or absence of a fetal heart rate at the onset of labor to distinguish between intrapartum and prepartum stillbirth, which would have helped to understand the importance of delays in accessing care or after admission. Second, a small proportion of delivery outcomes was imputed. Although the reported results were similar to those obtained from the same analysis of observed data, the true values may be different. Furthermore, imputed data values will vary slightly depending on the number of imputations conducted, which might reduce the replicability of the analysis. Last, similar to other studies from resource‐limited settings,^25^ some neonates that were born alive but died shortly thereafter might have been classified as stillborn, affecting the number of stillbirths recorded in the study.

In conclusion, the risk of stillbirth was found to be higher among patients who experienced intrapartum near‐miss events than among patients who experienced intrapartum complications without near‐miss events. Prompt action to prevent the development of organ dysfunction in the mother, coupled with early management of complications, has the potential to save many newborns.

## Author Contributions

AH and CH conceived the study. In Tanzania, FA, CH, and ABP supervised the trial and data collection with support from JLM. In Uganda, SA, CH, and FK supervised the trial and data collection with support from JLM. AH performed statistical analysis with support from GM and CH. AH drafted the manuscript. All authors revised and approved the final manuscript.

## Conflicts of Interest

JLM is employed by the International Federation of Gynecology and Obstetrics (FIGO). The authors have no other conflicts of interest.

## Supporting information


**Figure S1.** Conceptual framework.

**Figure S2.** Rates of stillbirth per 1000 complicated deliveries and 95% confidence intervals by risk group in Tanzania and Uganda (observed data only).

**Figure S3.** Number of women who had organ dysfunctions and timing of occurrence in Tanzania (top) and Uganda (bottom). Data are shown for women in the intrapartum near‐miss group.

**Table S1.** Categorization of risk groups.

**Table S2.** Comparison of women who did and did not have delivery outcome data available.

**Table S3.** Underlying causes, and maternal and fetal risk factors by severity of obstetric complications in Tanzania and Uganda (observed data only).

**Table S4.** Stillbirth rates per 1000 complicated deliveries by maternal, delivery, and fetal factors (observed data only).

**Table S5.** Stillbirth rates stratified by delivery mode, gestational age, and place of delivery in Tanzania (top) and Uganda (bottom) (observed data only).

**Table S6.** Risk of stillbirth in obstetric complication groups in Tanzania and Uganda.

**File S1.** Data collection tool.
