# Supplementary material for: Disentangling the contributions of maternal and fetal factors to estimate stillbirth risks for intrapartum adverse events in Tanzania and Uganda
Source: Int J Gynaecol Obstet. 2018 Oct 26;144(1):37–48. doi: 10.1002/ijgo.12689 (PMC7379231; doi:10.1002/ijgo.12689)
Supplement: Supplementary file 1 — Figure S1. Conceptual framework. [file IJGO-144-37-s001.pptx]

## Slide 1
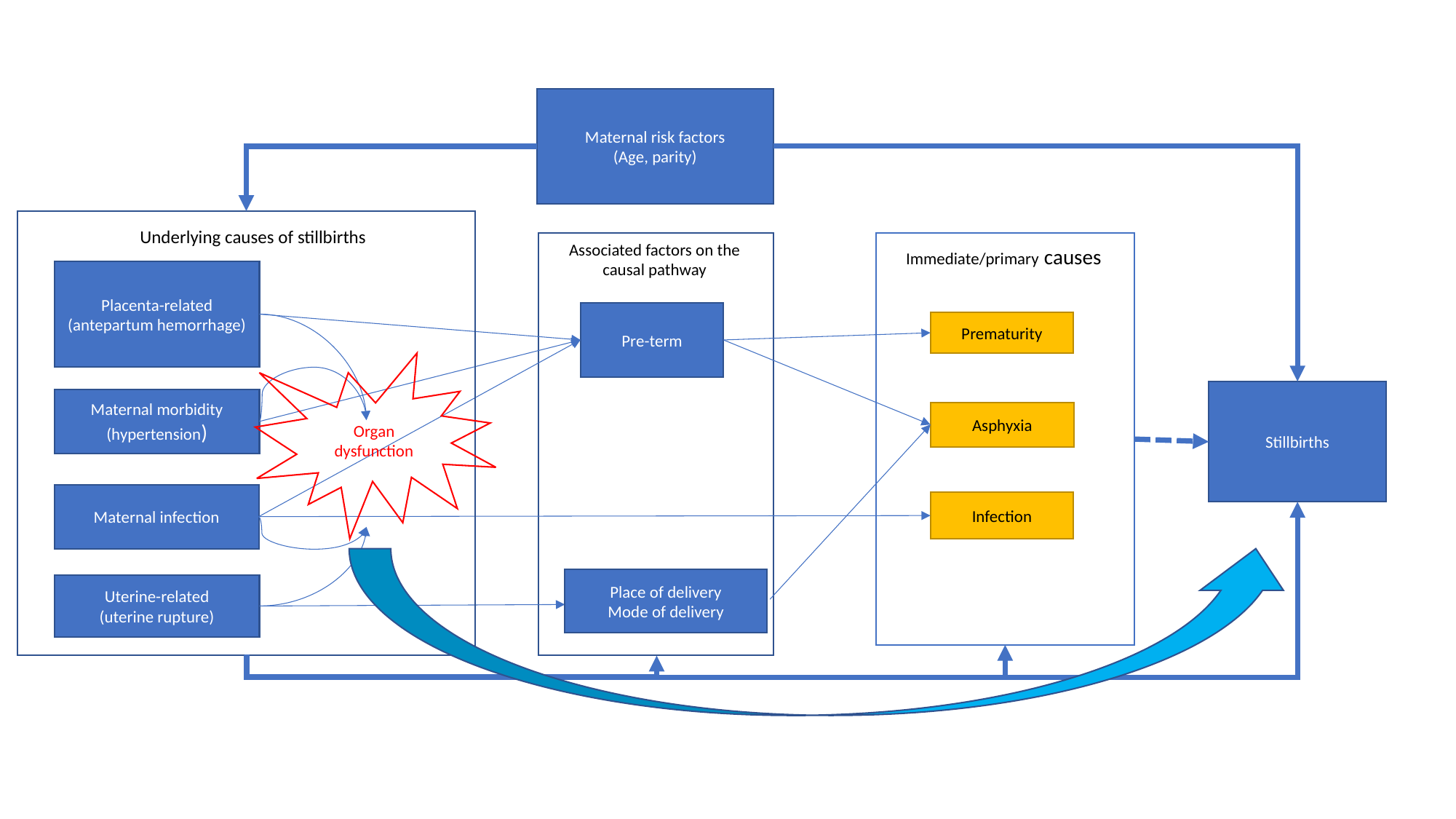

Maternal risk factors
(Age, parity)
Underlying causes of stillbirths
Associated factors on the causal pathway
Immediate/primary causes
Placenta-related
(antepartum hemorrhage)
Pre-term
Prematurity
Organ dysfunction
Stillbirths
Maternal morbidity
(hypertension)
Asphyxia
Maternal infection
Infection
Place of delivery
Mode of delivery
Uterine-related
(uterine rupture)
