# Supplementary figures and images for: Disentangling the contributions of maternal and fetal factors to estimate stillbirth risks for intrapartum adverse events in Tanzania and Uganda
Source: Int J Gynaecol Obstet. 2018 Oct 26;144(1):37–48. doi: 10.1002/ijgo.12689 (PMC7379231; doi:10.1002/ijgo.12689)

## Slide 1
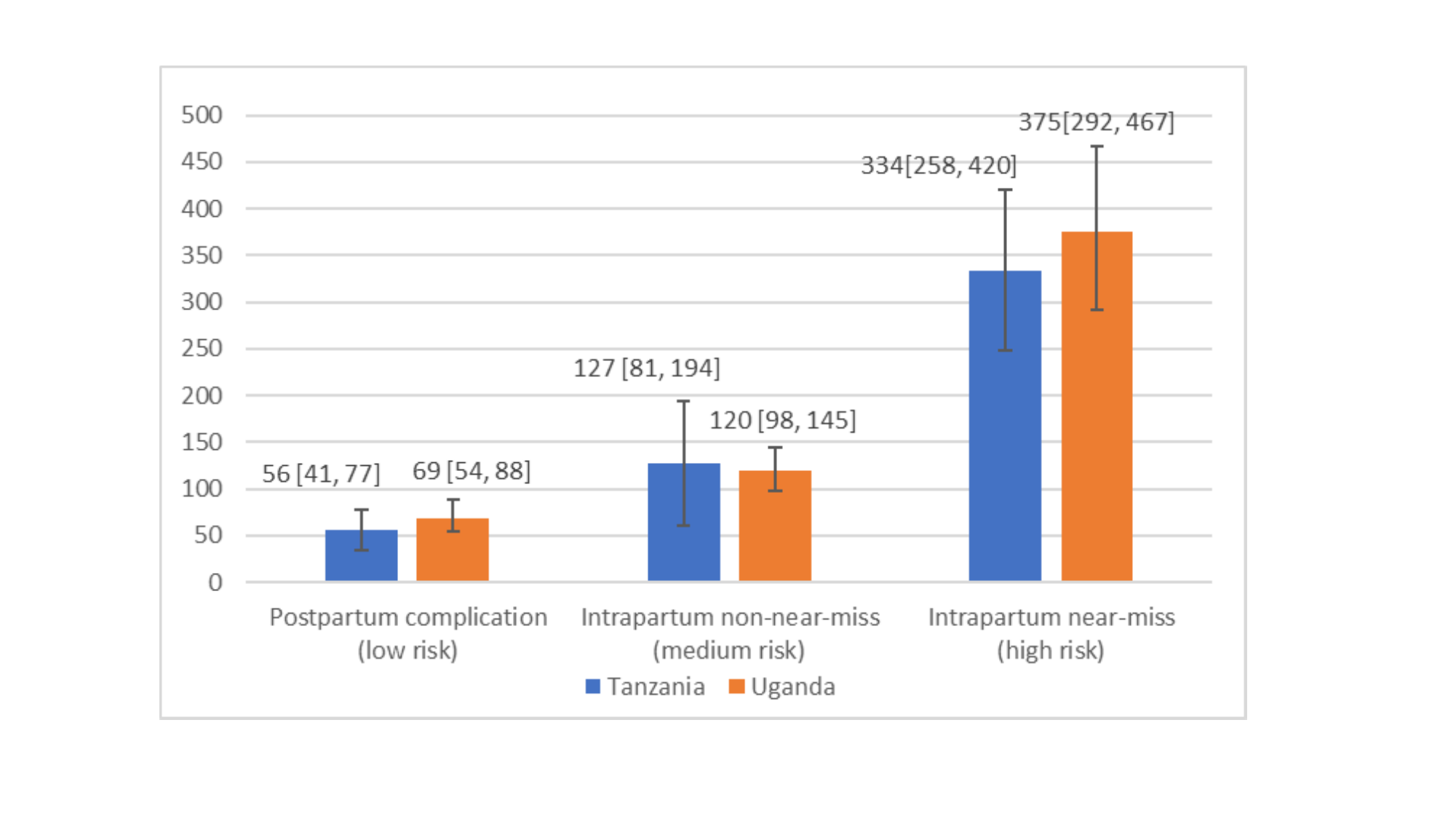

Supplement: Supplementary file 2 — Figure S2. Rates of stillbirth per 1000 complicated deliveries and 95% confidence intervals by risk group in Tanzania and Uganda (observed data only). [file IJGO-144-37-s002.pptx]
