# Supplementary material for: Disentangling the contributions of maternal and fetal factors to estimate stillbirth risks for intrapartum adverse events in Tanzania and Uganda
Source: Int J Gynaecol Obstet. 2018 Oct 26;144(1):37–48. doi: 10.1002/ijgo.12689 (PMC7379231; doi:10.1002/ijgo.12689)
Supplement: Supplementary file 3 — Figure S3. Number of women who had organ dysfunctions and timing of occurrence in Tanzania (top) and Uganda (bottom). Data are shown for women in the intrapartum near‐miss group. [file IJGO-144-37-s003.pptx]

## Slide 1
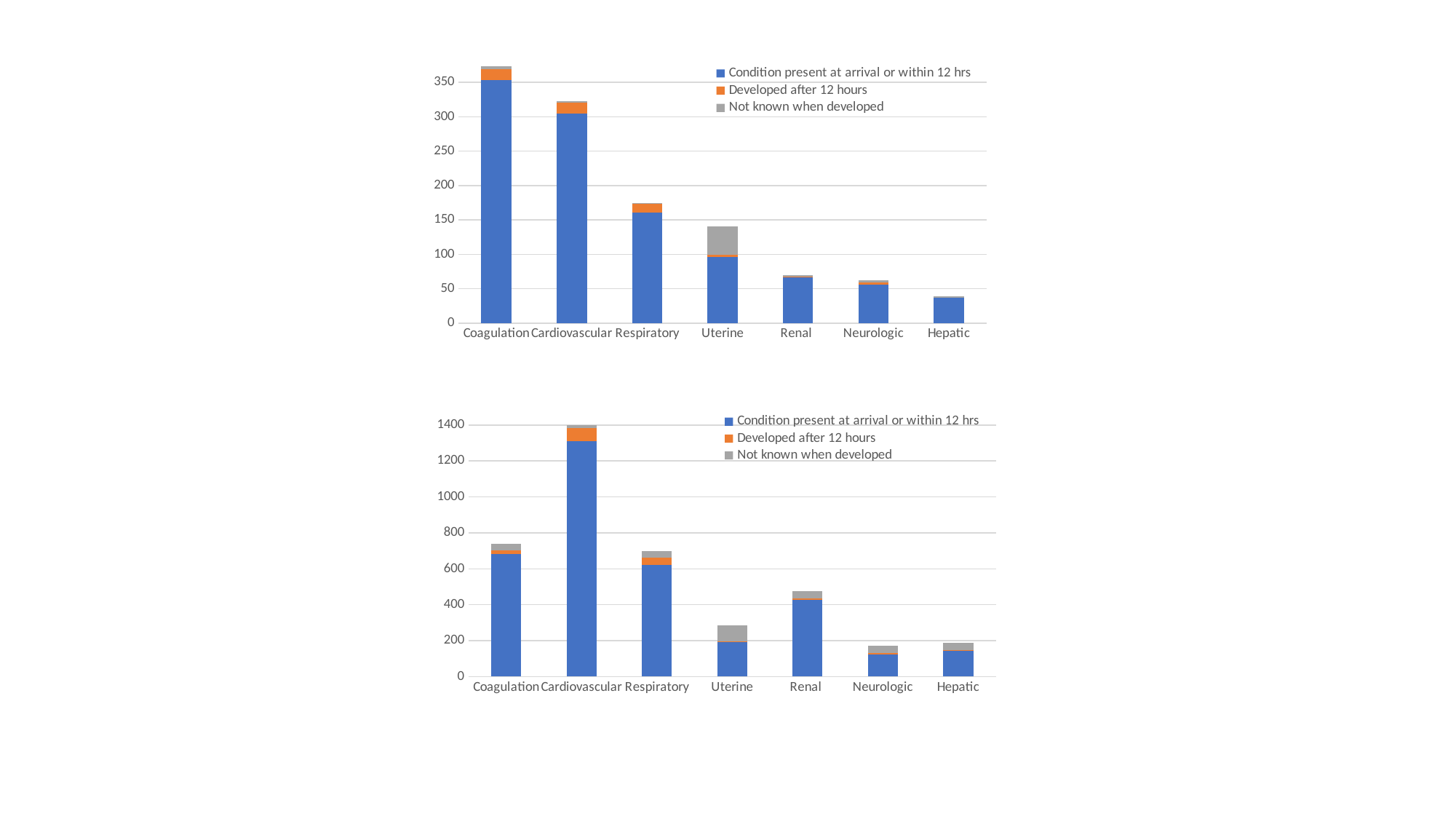

### Chart
| Category | Condition present at arrival or within 12 hrs | Developed after 12 hours | Not known when developed |
|---|---|---|---|
| Coagulation | 353.0 | 16.0 | 4.0 |
| Cardiovascular | 305.0 | 16.0 | 2.0 |
| Respiratory | 161.0 | 12.0 | 2.0 |
| Uterine | 96.0 | 3.0 | 42.0 |
| Renal | 66.0 | 2.0 | 2.0 |
| Neurologic | 56.0 | 3.0 | 3.0 |
| Hepatic | 37.0 | 0.0 | 2.0 |
### Chart
| Category | Condition present at arrival or within 12 hrs | Developed after 12 hours | Not known when developed |
|---|---|---|---|
| Coagulation | 682.0 | 20.0 | 37.0 |
| Cardiovascular | 1309.0 | 72.0 | 30.0 |
| Respiratory | 622.0 | 42.0 | 36.0 |
| Uterine | 190.0 | 5.0 | 91.0 |
| Renal | 426.0 | 10.0 | 39.0 |
| Neurologic | 125.0 | 8.0 | 40.0 |
| Hepatic | 144.0 | 4.0 | 40.0 |
