# Supplementary material for: Disentangling the contributions of maternal and fetal factors to estimate stillbirth risks for intrapartum adverse events in Tanzania and Uganda
Source: Int J Gynaecol Obstet. 2018 Oct 26;144(1):37–48. doi: 10.1002/ijgo.12689 (PMC7379231; doi:10.1002/ijgo.12689)
Supplement: Supplementary file 4 — Table S1. Categorization of risk groups. [file IJGO-144-37-s004.docx]

Table S1 Categorization of risk groups.

| Risk groups | Criteria |
| --- | --- |
| Low risk group (postpartum complications) | Experienced PPH AND NOT experiencing any antepartum or intrapartum complications |
| Medium risk group (intrapartum non-near-miss complications) | Experienced antepartum or intrapartum complications AND NOT experiencing organ dysfunction AND NOT having any management-based severity criteria (blood transfusion, hysterectomy) |
| High risk group (intrapartum near-miss complications) | Experienced antepartum or intrapartum complications AND experiencing organ dysfunction OR having management-based severity criteria (blood transfusion, hysterectomy) |
