# Supplementary material for: Disentangling the contributions of maternal and fetal factors to estimate stillbirth risks for intrapartum adverse events in Tanzania and Uganda
Source: Int J Gynaecol Obstet. 2018 Oct 26;144(1):37–48. doi: 10.1002/ijgo.12689 (PMC7379231; doi:10.1002/ijgo.12689)
Supplement: Supplementary file 5 — Table S2. Comparison of women who did and did not have delivery outcome data available. [file IJGO-144-37-s005.docx]

Table S2 Comparison of women who did and did not have delivery outcome data available

|  |  | Tanzania |  |  | Uganda |  |  |
| --- | --- | --- | --- | --- | --- | --- | --- |
|  |  | Outcome data not missing | Outcome data missing | p-value | Outcome data not missing | Outcome data missing | p-value |
| ***Age group*** | | N=3,086 | N=730 |  | N=7,846 | N=459 |  |
|  | <20 | 23 | 24 | 0.413 | 19 | 21 | 0.365 |
|  | 20-24 | 26 | 26 |  | 31 | 29 |  |
|  | 25-29 | 19 | 17 |  | 22 | 22 |  |
|  | 30-34 | 16 | 14 |  | 15 | 13 |  |
|  | 35-39 | 11 | 12 |  | 9 | 12 |  |
|  | >=40 | 6 | 7 |  | 3 | 3 |  |
| ***Parity*** | |  |  | 0.183 |  |  | 0.018 |
|  | Nullipara | 34 | 35 |  | 19 | 29 |  |
|  | para 1 | 19 | 17 |  | 23 | 17 |  |
|  | para 2 | 14 | 11 |  | 16 | 11 |  |
|  | para 3 or greater | 32 | 37 |  | 42 | 43 |  |
| ***Induction*** | |  |  | 0.153 |  |  | 0.211 |
|  | Labour induced | 5 | 11 |  | 10 | 4 |  |
|  | Not induced | 52 | 50 |  | 75 | 84 |  |
|  | Not recorded | 43 | 39 |  | 15 | 12 |  |
| ***Place of delivery*** | |  |  | 0.400 |  |  | <0.001 |
|  | hospital | 71 | 67 |  | 69 | 29 |  |
|  | health centre | 17 | 13 |  | 20 | 58 |  |
|  | other facility and referred | 3 | 3 |  | 7 | 5 |  |
|  | other | 10 | 17 |  | 5 | 8 |  |
| ***Delivery mode*** | |  |  | <0.001 |  |  | <0.001 |
|  | Vaginal | 64 | 61 |  | 49 | 22 |  |
|  | C-section/surgery | 35 | 26 |  | 50 | 6 |  |
|  | Unknown | 1 | 13 |  | 1 | 72 |  |
| ***Gestational age*** | |  |  | 0.290 |  |  | 0.010 |
|  | Preterm | 29 | 41 |  | 17 | 40 |  |
|  | Term | 67 | 56 |  | 71 | 51 |  |
|  | Postterm | 1 | 0 |  | 1 | 0 |  |
|  | missing | 3 | 3 |  | 12 | 8 |  |
| ***Birthweight*** | |  |  |  |  |  | 0.001 |
|  | Low birth | N/A |  |  | 17 | 42 |  |
|  | 2.5-4kg |  |  |  | 69 | 50 |  |
|  | >=4kg |  |  |  | 1 | 0 |  |
|  | missing |  |  |  | 13 | 8 |  |
| ***Risk groups*** | |  |  | <0.001 |  |  | <0.001 |
|  | Intrapartum near miss | 19 | 9 |  | 27 | 58 |  |
|  | Intrapartum non-near-miss complication | 33 | 56 |  | 19 | 28 |  |
|  | Postpartum complication | 48 | 35 |  | 54 | 14 |  |
