# Supplementary material for: Disentangling the contributions of maternal and fetal factors to estimate stillbirth risks for intrapartum adverse events in Tanzania and Uganda
Source: Int J Gynaecol Obstet. 2018 Oct 26;144(1):37–48. doi: 10.1002/ijgo.12689 (PMC7379231; doi:10.1002/ijgo.12689)
Supplement: Supplementary file 6 — Table S3. Underlying causes, and maternal and fetal risk factors by severity of obstetric complications in Tanzania and Uganda (observed data only). [file IJGO-144-37-s006.docx]

**Table S3** Underlying causes, and maternal and fetal risk factors by severity of obstetric complications in Tanzania and Uganda (observed data only)

|  |  | Tanzania Total | Postpartum complication | Intrapartum complications | Intrapartum near-miss | p-value | Uganda Total | Postpartum complication | Intrapartum complications | Intrapartum near-miss | p-value |
| --- | --- | --- | --- | --- | --- | --- | --- | --- | --- | --- | --- |
|  |  | N=3,086 | N=1,491 | N=1,005 | N=590 |  | N=7,846 | N=4,222 | N=1,515 | N=2,109 |  |
| ***Underlying causes*** | | (%) | (%) | (%) | (%) |  |  | (%) | (%) | (%) |  |
| ***Maternal complications*** | |  |  |  |  |  |  |  |  |  |  |
|  | Postpartum haemorrhage | 52 | 100 | 4 | 16 | <0.001 | 64 | 100 | 12 | 28 | <0.001 |
|  | Hypertensive disorders | 24 | 0 | 56 | 31 | <0.001 | 19 | 0 | 53 | 32 | <0.001 |
|  | Infection | 12 | 0 | 21 | 24 | <0.001 | 13 | 0 | 15 | 37 | <0.001 |
|  | Rupture of the uterus | 5 | 0 | 2 | 22 | <0.001 | 8 | 0 | 5 | 24 | <0.001 |
|  | Antepartum haemorrhage | 13 | 0 | 23 | 30 | <0.001 | 13 | 0 | 29 | 25 | <0.001 |
| ***Maternal risk factors*** | |  |  |  |  |  |  |  |  |  |  |
| ***Age*** | |  |  |  |  |  |  |  |  |  |  |
|  | <20 | 23 | 23 | 25 | 21 | 0.128 | 19 | 20 | 21 | 16 | 0.014 |
|  | 20-24 | 26 | 23 | 27 | 24 |  | 31 | 32 | 30 | 31 |  |
|  | 25-29 | 19 | 18 | 19 | 20 |  | 22 | 22 | 23 | 22 |  |
|  | 30-34 | 16 | 17 | 14 | 15 |  | 15 | 14 | 15 | 17 |  |
|  | 35-39 | 11 | 11 | 9 | 14 |  | 9 | 9 | 9 | 10 |  |
|  | >=40 | 6 | 6 | 5 | 7 |  | 3 | 2 | 3 | 3 |  |
|  | Median age (in years) | 25 | 25 | 24 | 26 | 0.002 | 24 | 24 | 24 | 25 | <0.001 |
| ***Parity*** | |  |  |  |  | 0.178 |  |  |  |  | 0.281 |
|  | Nulliparaous | 35 | 34 | 39 | 30 |  | 19 | 19 | 21 | 20 |  |
|  | Para 1 | 18 | 17 | 19 | 20 |  | 23 | 22 | 26 | 22 |  |
|  | Para 2 | 14 | 14 | 14 | 15 |  | 16 | 17 | 15 | 16 |  |
|  | Para 3 or greater | 33 | 35 | 28 | 36 |  | 42 | 43 | 38 | 43 |  |
| ***Delivery factors*** | |  |  |  |  |  |  |  |  |  |  |
| ***Mode of delivery*** | |  |  |  |  |  |  |  |  |  |  |
|  | Vaginal | 64 | 86 | 53 | 30 | <0.001 | 49 | 70 | 29 | 23 | <0.001 |
|  | C-section/surgery | 35 | 14 | 46 | 69 |  | 50 | 30 | 70 | 75 |  |
|  | Unknown | 1 | 0 | 1 | 1 |  | 1 | <1 | 1 | 1 |  |
| ***Place of delivery*** | |  |  |  |  |  |  |  |  |  |  |
|  | At study hospital | 71 | 63 | 78 | 77 | 0.011 | 69 | 56 | 86 | 81 | <0.001 |
|  | At study health centre | 17 | 25 | 7 | 13 |  | 20 | 29 | 8 | 11 |  |
|  | Other facility and referred | 3 | 4 | 1 | 3 |  | 7 | 9 | 3 | 5 |  |
|  | Other | 10 | 8 | 14 | 7 |  | 5 | 6 | 3 | 4 |  |
| ***Gestational weeks*** | |  |  |  |  |  |  |  |  |  |  |
|  | Preterm births (<37wks) | 29 | 22 | 35 | 37 | 0.037 | 17 | 11 | 25 | 25 | <0.001 |
|  | Term | 67 | 74 | 63 | 58 |  | 69 | 76 | 65 | 60 |  |
|  | Post term | 1 | 1 | 1 | 1 |  | 1 | 1 | <1 | 1 |  |
|  | Missing | 3 | 2 | 1 | 5 |  | 13 | 13 | 10 | 15 |  |
| ***Birthweight*** | |  |  |  |  |  |  |  |  |  |  |
|  | Low birthweight | N/A |  |  |  |  | 13 | 7 | 20 | 19 | <0.001 |
|  | 2.5-4kg |  |  |  |  |  | 67 | 74 | 62 | 56 |  |
|  | >=4kg |  |  |  |  |  | 6 | 77 | 5 | 5 |  |
|  | Missing |  |  |  |  |  | 15 | 12 | 14 | 21 |  |
