# Supplementary material for: Disentangling the contributions of maternal and fetal factors to estimate stillbirth risks for intrapartum adverse events in Tanzania and Uganda
Source: Int J Gynaecol Obstet. 2018 Oct 26;144(1):37–48. doi: 10.1002/ijgo.12689 (PMC7379231; doi:10.1002/ijgo.12689)
Supplement: Supplementary file 7 — Table S4. Stillbirth rates per 1000 complicated deliveries by maternal, delivery, and fetal factors (observed data only). [file IJGO-144-37-s007.docx]

**Table S4** Stillbirth rates per 1000 complicated deliveries by maternal, delivery, and fetal factors (observed data only).

|  |  | Tanzania |  |  |  | Uganda |  |  |  |
| --- | --- | --- | --- | --- | --- | --- | --- | --- | --- |
|  |  | All births (N) | Live births (%) | Stillbirths (%) | stillbirth rates per 1000 births | All births (N) | Live births (%) | Stillbirths (%) | stillbirth rates per 1000 births |
| ***Maternal factors*** | |  |  |  |  |  |  |  |  |
| ***Age*** | | N=3,086 | 100% | 100% |  | N=7,846 | 100% | 100% |  |
|  | <20 | 714 | 24.4 | 14.9 | 85.4 [60.9, 118.6] | 1,503 | 20.2 | 13.3 | 99.8 [79.7, 124.3] |
|  | 20-24 | 788 | 25.8 | 23.7 | 123.1 [91.3, 164.0] | 2,467 | 31.8 | 29.2 | 133.8 [109.2, 162.8] |
|  | 25-29 | 580 | 18.4 | 21.3 | 150.0 [109.0, 202.8] | 1,754 | 22.5 | 21.4 | 138.0 [107.4, 175.5] |
|  | 30-34 | 487 | 15.5 | 17.4 | 145.8 [97.0, 213.3] | 1,184 | 14.2 | 20.5 | 195.9 [156.7, 242.2] |
|  | 35-39 | 343 | 10.6 | 14.7 | 174.9 [126.8, 236.3] | 730 | 8.8 | 12.1 | 187.7 [150.7, 231.3] |
|  | >=40 | 174 | 5.3 | 8.1 | 189.7 [122.9, 281.0] | 208 | 2.5 | 3.5 | 192.3 [142.0, 255.2] |
| ***Parity*** | |  |  |  |  |  |  |  |  |
|  | Nulliparaous | 1077 | 36.5 | 24.5 | 92.9 [70.9, 120.7] | 1,525 | 20.6 | 12.7 | 94.4 [74.1, 119.7] |
|  | Para 1 | 555 | 17.8 | 19.1 | 140.5 [95.0, 203.0] | 1,789 | 23.5 | 18.6 | 117.4 [88.9, 153.5] |
|  | Para 2 | 443 | 14.2 | 15.2 | 140.0 [93.5, 204.2] | 1,259 | 16.1 | 15.9 | 143.0 [115.3, 175.9] |
|  | Para 3 or greater | 1011 | 31.5 | 41.3 | 167.2 [120.9, 226.6] | 3,273 | 39.9 | 52.8 | 182.4 [151.3, 218.2] |
| ***Delivery factors*** | |  |  |  |  |  |  |  |  |
| *Mode of delivery* | |  |  |  |  |  |  |  |  |
|  | Vaginal | 1988 | 67.1 | 46.9 | 96.6 [74.6, 124.2] | 3,867 | 51.7 | 35.3 | 103.2 [86.9, 122.2] |
|  | C-section/surgery | 1081 | 32.5 | 51.3 | 194.3 [140.7, 262.1] | 3,932 | 47.8 | 64.0 | 184.1 [142.9, 234.0] |
|  | Unknown | 17 | 0.4 | 1.7 | 411.8 [175.7, 696.9] | 47 | 0.6 | 0.7 | 170.2 [61.8, 389.7] |
| *Place of delivery* | |  |  |  |  |  |  |  |  |
|  | At study hospital | 2178 | 69.2 | 79.7 | 149.7 [111.3, 198.3] | 5,383 | 67.0 | 78.2 | 164.2 [131.6, 203.0] |
|  | At study health centre | 512 | 17.7 | 9.5 | 76.2 [42.1, 133.9] | 1,564 | 21.6 | 10.0 | 72.3 [52.9, 97.9] |
|  | Other facility and referred | 88 | 2.9 | 2.7 | 125.0 [81.0, 188.0] | 535 | 6.6 | 8.3 | 175.7 [138.9, 220.6] |
|  | Other | 308 | 10.3 | 8.1 | 107.1 [48.5, 220.5] | 364 | 4.8 | 3.5 | 109.9 [78.8, 151.2] |
| ***Foetal factors*** | |  |  |  |  |  |  |  |  |
| Gestational weeks | |  |  |  |  |  |  |  |  |
|  | Preterm births (<37wks) | 901 | 26 | 50 | 225.3 [182.5, 274.8] | 1,363 | 15.7 | 28.8 | 237.7 [196.7, 281.7] |
|  | Term | 2077 | 71 | 45 | 89.6 [64.4, 123.2] | 5,437 | 71.3 | 55.8 | 116.4 [89.6, 150.0] |
|  | Post term | 28 | 1 | 0 | 71.4 [23.4, 198.3] | 42 | 0.6 | 0.2 | 47.6 [7.6, 246.5] |
|  | Missing | 80 | 2 | 4 | 225.0 [117.6, 387.4] | 1,004 | 12.4 | 15.2 | 171.3 [138.7, 209.8] |
| Birthweight | |  |  |  |  |  |  |  |  |
|  | Low birthweight | N/A |  |  |  | 986 | 11.6 | 18.3 | 209.9 [179.4, 244.2] |
|  | 2.5-4kg |  |  |  |  | 5,227 | 70.3 | 44.4 | 96.0 [74.0, 123.7] |
|  | >=4kg |  |  |  |  | 474 | 6.1 | 5.8 | 139.2 [97.2, 195.5] |
|  | Missing |  |  |  |  | 1,159 | 12.0 | 31.5 | 307.2 [258.5, 360.5] |
