# Supplementary material for: Disentangling the contributions of maternal and fetal factors to estimate stillbirth risks for intrapartum adverse events in Tanzania and Uganda
Source: Int J Gynaecol Obstet. 2018 Oct 26;144(1):37–48. doi: 10.1002/ijgo.12689 (PMC7379231; doi:10.1002/ijgo.12689)
Supplement: Supplementary file 8 — Table S5. Stillbirth rates stratified by delivery mode, gestational age, and place of delivery in Tanzania (top) and Uganda (bottom) (observed data only). [file IJGO-144-37-s008.docx]

**Table S5** Stillbirth rates stratified by delivery mode, gestational age, and place of delivery in Tanzania (top) and Uganda (bottom) (observed data only).

|  |  | Postpartum complications  (low risk) | | | Intrapartum non-near-miss group  (medium risk) | | | Intrapartum near-miss group  (high risk) | | |
| --- | --- | --- | --- | --- | --- | --- | --- | --- | --- | --- |
|  |  | Stillbirth rate [95%CI] | p-value | aPR* | Stillbirth rate [95%CI] | p-value | aPR* | Stillbirth rate [95%CI] | p-value | aPR* |
| ***Gestational age*** | | |  |  |  |  |  |  |  |  |
|  | Preterm | 98.5 [65.3, 146.0] | 0.003 | 2.4 [1.5, 3.8] | 223.5 [165.4, 294.7] | 0.002 | 3.1 [2.1, 4.5] | 424.0 [332.3, 521.2] | 0.002 | 1.8 [1.3, 2.5] |
|  | Term | 41.8 [27.5, 63.1] | Ref | 1.0 | 72.3 [38.1, 133.2] | Ref | 1.0 | 276.5 [198.5, 370.9] | Ref | 1.0 |
| ***Delivery mode*** | | |  |  |  |  |  |  |  |  |
|  | C-section | 42.3 [19.0, 91.3] | 0.384 | 0.7 [0.4, 1.5] | 110.4 [68.4, 173.3] | 0.001 | 0.5 [0.3, 0.8] | 369.5 [278.8, 470.3] | 0.238 | 0.8 [0.5, 1.2] |
|  | Vaginal | 57.3 [41.4, 78.6] | Ref | 1.0 | 143.4 [85.9, 229.8] | Ref | 1.0 | 238.6 [158.2, 343.4] | Ref | 1.0 |
| ***Place of delivery*** | | |  |  |  |  |  |  |  |  |
|  | Hospital | 51.0 [35.2, 73.4] | Ref | 1.0 | 140.8 [94.4, 205.0] | Ref | 1.0 | 368.4 [280.0, 466.7] | Ref | 1.0 |
|  | Clinic | 35.2 [17.2, 70.8] | 0.352 | 0.6 [0.3, 1.1] | 101.4 [41.9, 225.8] | 0.474 | 0.7 [0.3, 1.6] | 256.8 [103.3, 508.9] | 0.363 | 0.9 [0.5, 1.4] |

|  | | Postpartum complications  (low risk) | | | Intrapartum non-near miss group   (medium risk) | | | | Intrapartum near-miss group  (high risk) | | | |
| --- | --- | --- | --- | --- | --- | --- | --- | --- | --- | --- | --- | --- |
|  |  | Stillbirth rate [95%CI] | p-value | aPR* | Stillbirth rate [95%CI] | p-value | aPR* | Stillbirth rate [95%CI] | | p-value | aPR* |  |
| ***Gestational age*** | |  |  |  |  |  |  |  | |  |  |  |
|  | Preterm | 181.2 [129.1, 248.4] | <0.001 | 4.0[3.1, 5.3] | 213.9 [175.5, 258.1] | <0.001 | 2.6[1.8, 3.5] | 300.9 [219.8, 396.7] | | 0.033 | 1.2 [1.0, 1.5] |  |
|  | Term | 45.2 [35.0, 59.8] |  | 1.0 | 87.2 [65.2, 115.8] | Ref | 1.0 | 322.3 [248.0, 406.9] | | Ref | 1.0 |  |
| ***Delivery mode*** | |  |  |  |  |  |  |  | |  |  |  |
|  | C-section | 70.1 [46.1, 105.4] | 0.726 | 1.0 [0.8, 1.3] | 99.6 [78.7, 125.3] | <0.001 | 0.4 [0.3, 0.6] | 334.0 [257.8, 419.9] | | 0.028 | 0.8 [0.6, 1.0] |  |
|  | Vaginal | 66.6 [54.9, 80.6] |  | 1.0 | 167.8 [139.5, 200.6] |  | 1.0 | 262.2 [212.2, 319.2] | |  | 1.0 |  |
| ***Place of delivery*** | |  |  |  |  |  |  |  | |  |  |  |
|  | Hospital | 72.3 [54.4, 95.5] | Ref | 1.0 | 116.1 [96.1, 139.5] | Ref | 1.0 | 330.4 [255.8, 414.6] | | Ref | 1.0 |  |
|  | Clinic | 37.9 [27.4, 52.0] | <0.001 | 0.5 [0.4, 0.7] | 148.4 [74.0, 275.5] | 0.706 | 0.9 [0.6, 1.5] | 216.2 [134.8, 328.2] | | 0.150 | 0.8 [0.6, 1.1] |  |

*Adjusted for age, parity, and complication types; abbreviation: aPR = adjusted prevalence ratio
