# Supplementary material for: Disentangling the contributions of maternal and fetal factors to estimate stillbirth risks for intrapartum adverse events in Tanzania and Uganda
Source: Int J Gynaecol Obstet. 2018 Oct 26;144(1):37–48. doi: 10.1002/ijgo.12689 (PMC7379231; doi:10.1002/ijgo.12689)
Supplement: Supplementary file 9 — Table S6. Risk of stillbirth in obstetric complication groups in Tanzania and Uganda. [file IJGO-144-37-s009.docx]

Table S6 Risk of stillbirth in obstetric complication groups in Tanzania and Uganda

|  | Tanzania |  |  | Uganda |  |  |  |
| --- | --- | --- | --- | --- | --- | --- | --- |
|  | PR | aPR | p-value^a^ | PR | aPR | p-value^a^ |  |
| Intrapartum near-miss^b^ | 6.06 [4.51, 8.16] | 4.55 [2.94, 7.04] | <0.001 | 4.67 [3.95, 5.51] | 3.73 [2.86, 4.88] | <0.001 |  |
| Intrapartum non-near-miss | 2.39 [1.58, 3.66] | 2.62 [1.49, 4.61] | <0.001 | 1.82 [1.40, 2.35] | 1.92 [1.43, 2.58] | <0.001 |  |
| Postpartum complications | 1.0 | 1.0 | Ref | 1.0 | 1.0 | Ref |  |
| ^a^ adjusted for age, parity and complication types  ^b^ Near-miss includes women having intrapartum complications with organ dysfunction  Abbreviations: PR = prevalence ratio; aPR=adjusted prevalence ratio | | | | | | | |
