# Supplementary material for: Disentangling the contributions of maternal and fetal factors to estimate stillbirth risks for intrapartum adverse events in Tanzania and Uganda
Source: Int J Gynaecol Obstet. 2018 Oct 26;144(1):37–48. doi: 10.1002/ijgo.12689 (PMC7379231; doi:10.1002/ijgo.12689)
Supplement: Supplementary file 10 — File S1. Data collection tool. [file IJGO-144-37-s010.docx]

File S1 Data collection tool

**Near Miss data collection tool HMS-BAB**

**
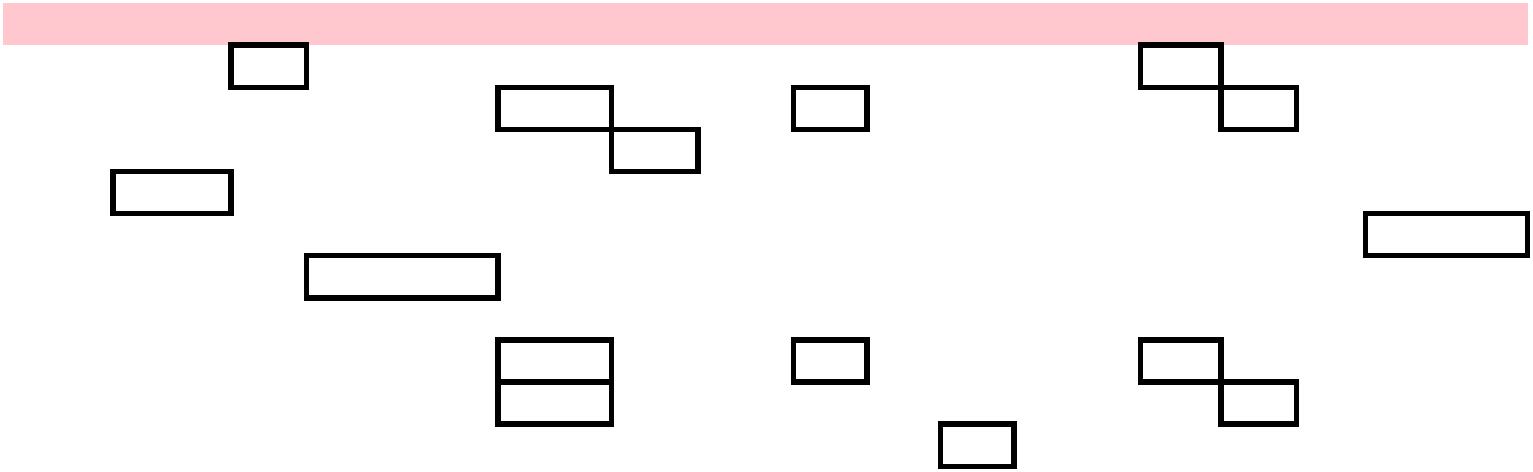
**

**Module 1 "Birth Recording"**

**Facility code** **Enter the admission number as available from records"**

**Mode of delivery** **spontaneous** **vacuum** **Caesarean section**

**Any induction /augmentation of labour?** **Cephalic Breech** **Other**

**Parity**

**What is the HIV status** **positive/negative/not recorded** **Gestational age in weeks**

**Number of babies**

**Report the babies outcome (if twins or triplets the outcome of the first twin)**

**Babies outcome?** **alive** **mac stillbirth** **fresh stillbirth**

**Oxytocine given?**

**Estimate the blood loss in terms of kangas soaked:**

**No of Kangas soaked**

**Any Complications recorded?**

**
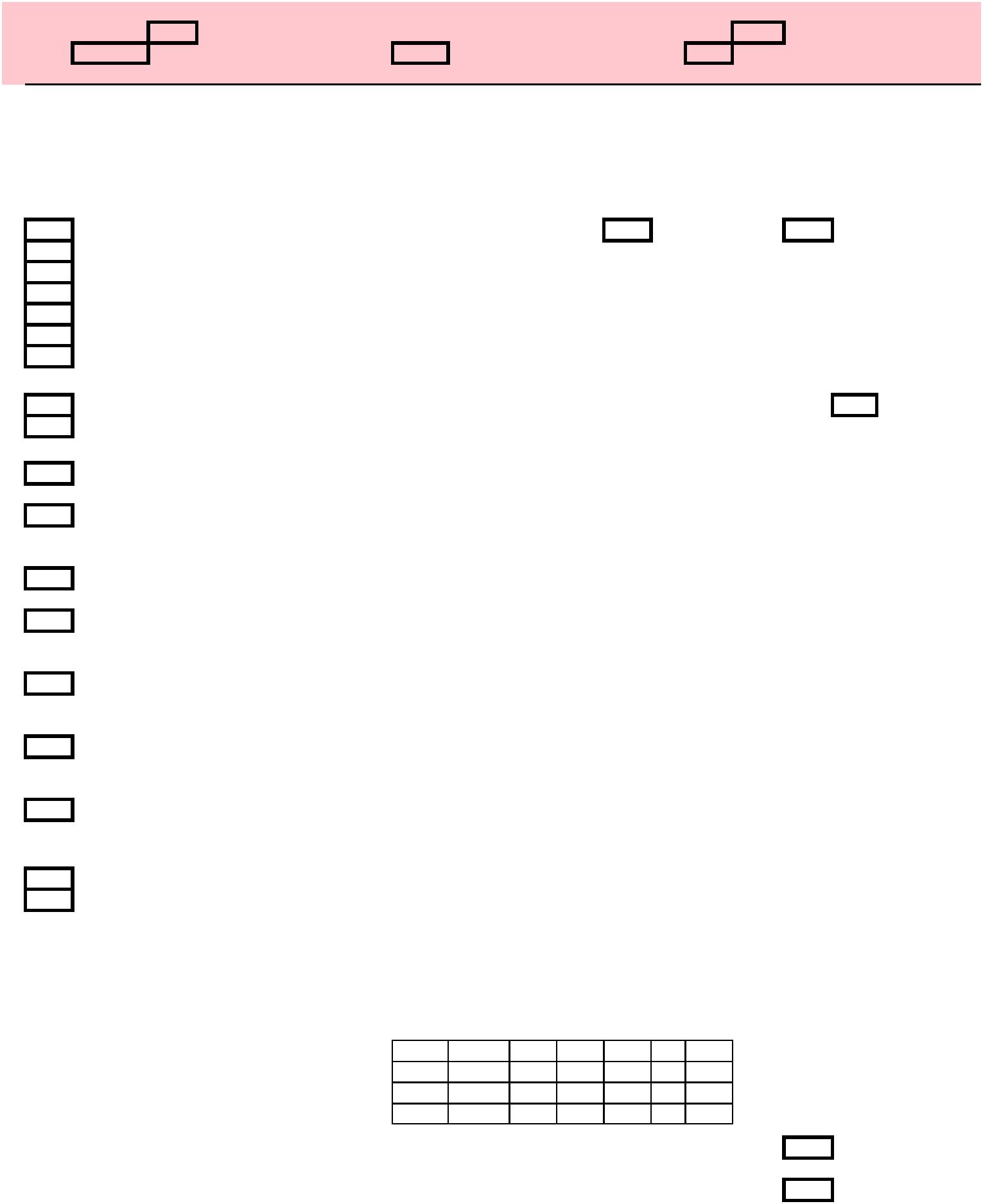
Module 2 (Screening questions)**

**Q: Facility code** **Enter the admission number as available from records**

**Age** **Parity**

**Place of living: District** **Ward**

**Gravidity**

**Village**

**Screening questions**

**Explanation: "For each of the question (1-4) below, please answer 1) if the condition was present at arrival or within 12 hours,**

1. **developped after 12 hours, or 3) if it is not known when it developed. Please enter 0 if the condition was not present**

**In the question 1 to 4, please specify**

**Please enter 0 if the condition was not present during the stay 1. Severe complications / potentially life-threatening conditions**

| **A 0** | **Any postpartum haemorrhage** | **>500ml** | **>1000ml** |
| --- | --- | --- | --- |
| **A 1** | **Severe preecplampsia** |  |  |
| **A 2** | **Severe eclampsia** |  |  |
| **A 3** | **Sepsis or severe infections** |  |  |
| **A 4** | **Ruptured uterus** |  |  |
| **A 5** | **Severe abortion complications** |  |  |
| **A 6** | **Severe antepartum haemorrhage** |  |  |
| **2. Critical interventions or intensive care unit admissions** | |  |  |
| **B 1** | **use of blood products (includes any blood transfusion)** | | **How many:** |
| **B 3** | **Laparotomy** |  |  |
| **3. Organ dysfunction /life threatening condition** | |  |  |
| **C 0** | **Cardiovascular disfunction** |  |  |
|  | **(shock, cardio-pulmonary rescucitation, severe acidosis- ph<7.1)** | |  |
| **C 1** | **Respiratory dysfunction** |  |  |
|  | **(acute cyanosis, gasping, severe tachypnoe -respiratory rate >40bpm-, severe brachypnoe,** | | |
|  | **severe hypoxemia (O2 saturation < 90% for > 1 hour)** | |  |
| **C 2** | **Renal dysfuntion** |  |  |
|  | **(oliguria not responsive to fluids or diuretics, creatinin >300umol/ml or 3.5mg/dl)** | | |
| **C 3** | **Coagulation / hematologic dysfuncyion** |  |  |
|  | **(failure to form clots, massive transfusion of blood cells, or** | |  |
|  | **severe acute thrombocytopenia (<50,000 platlets/ml)** | |  |
| **C 4** | **Hepatic dysfunction** |  |  |
|  | **(Jaundice in the presence of pre-eclampsia,** |  |  |
|  | **severe acute hyperbilirubinemia - >100umol/L or > 6mg/dL)** | |  |
| **C 5** | **Neurologic dysfunction** |  |  |
|  | **(prolonged uncounsciousness / coma (lasting >12 hours), stroke, status epilepticus /** | | |
|  | **uncontrolled fits, total paralysis)** |  |  |
| **C 6** | **Uterine disfunction /hysterectomy** |  |  |
|  | **(hemorrhage or infection leading to hysterectomy)** | |  |

**4. Maternal deaths**

**D 0** **Deaths during pregnancy or within 42 days after termination of pregnancy**

**D 1** **Death after 42 days of termination of pregnancy**

**Pleae note:**

1. **If you answered "1, 2" or "3" to any of the questions 1 to 4, go to questions 5**
2. **If you answered "0" to all the questions 1 to 4, the women is not eligible, do not answer any other questions (5-14)**

**iii In case you doubt on questions 1 to 4, consult the attending physician/midwife**

1. **In the question 5-14, if information is not available, unkown or not applicable fill with "9" Maternal and Perinatal Information**

|  | **d** | **d** | **m** | **m** | **20** | **y** | **y** |
| --- | --- | --- | --- | --- | --- | --- | --- |
| **5.** | **Date of hospital/facility admission** |  |  |  | **20** |  |  |
| **6.** | **Date of delivery or uterin evacuation** |  |  |  | **20** |  |  |
| **7.** | **Date of hospital discharge or deaths** |  |  |  | **20** |  |  |

**7 b: If women had haemorrhage or blood transfusion please also entre the Hb at discharge (g/dl)**

| **8. Please specify the final mode of delivery / end of pregnancy** | | | | **E 3** |
| --- | --- | --- | --- | --- |
| **1 =** | **Vaginal delivery** | **5=** | **Vacuum extraction** |  |
| **2 =** | **Ceasarean section** | **6=** | **Laparatomy for ectopic pregnancy** |  |
| **3 =** | **Complete abortion** | **7=** | **Laperatomy for ruptured uterus** |  |
| **4 =** | **Curretage/MVA** | **8=** | **Women discharged or died still pregnant** |  |
|  |  | **9=** | **Unknown** |  |

| **Module 3 "Nearmiss case"** |  |  |  |  |
| --- | --- | --- | --- | --- |
| **Facility code** |  | **Admission number** | |  |
| **9. Please check in the records the best estimate of gestational age in completed weeks (obstetrical/neonatal) at:** | | | |  |
|  | **Delivery or abortion (not applicable if Q8="8")** | | | **E 4** |
| **Maternal deaths or hospital discharge (applicable if Q 8="8")** | | | | **E 5** |
| **10. Please indicate the vital status of the infant (if twins, indicate outcome of first twin) ; 0=Alive 1 = Dead** | | | |  |
| **at birth** |  | **E 6** | **if 1= fresh / mazerated stillbirth/not recorded** | |
| **after 24 hours** |  | **E 7** |  |  |
| **at hospital/facility discharge or 7th day of life** | | **E 8** |  |  |
| **Please give the birth weigh** | **birthweight** |  | **E 9** |  |


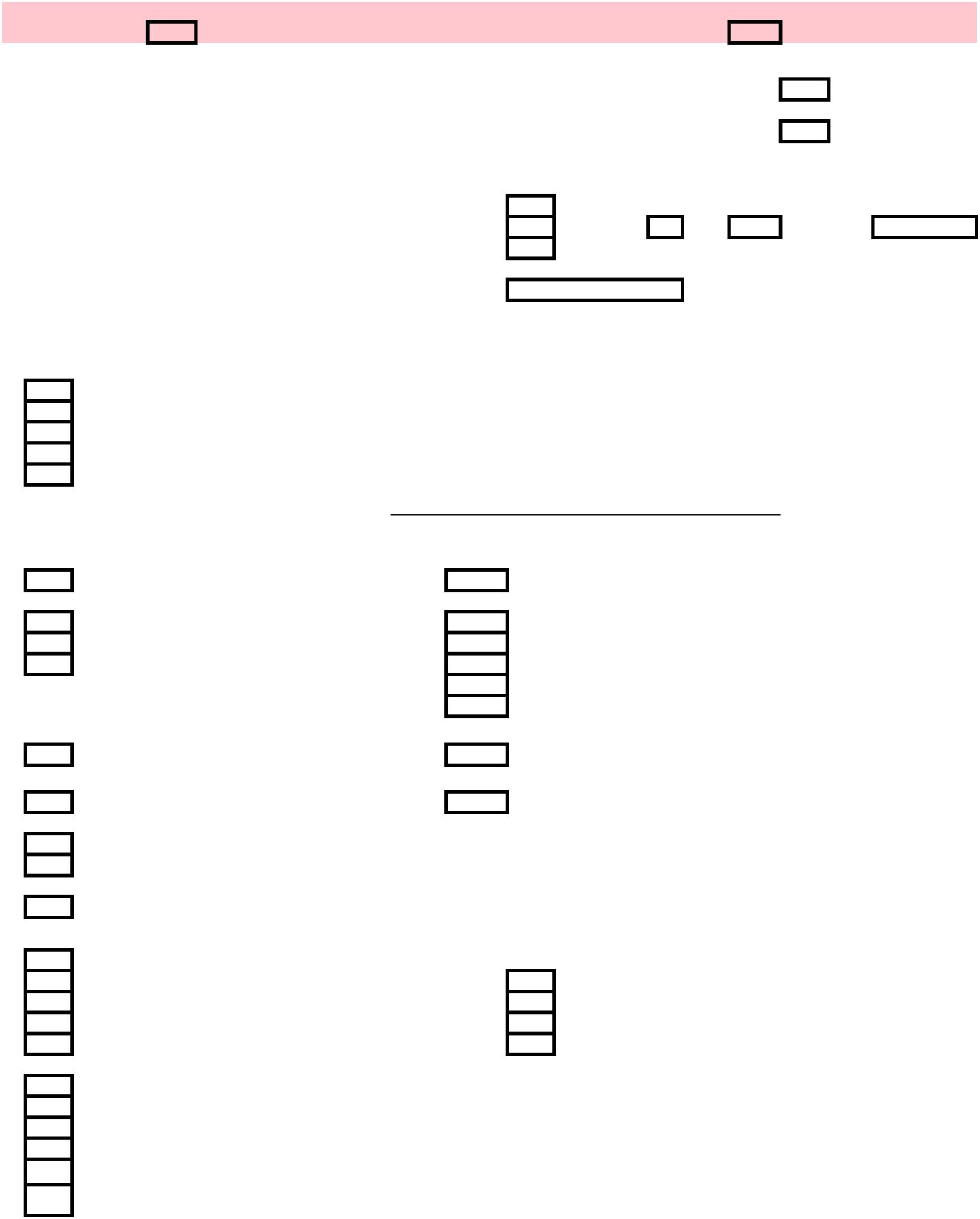


**Please try to check all forms to get the following information on process indicators**

**11. Please indicate whether any of the following applies concerning the conditions at arrival in the facility/ referral process,**

|  | **specify: 0 = no; 1 = yes** |
| --- | --- |
| **F 0** | **Delivery or abortion occured before arrival at facility** |
| **F 1** | **Delivery within 3 hours of arrival in the health facility** |
| **F 2** | **Laparatomy within 3 hours of hospital arrival or in another hospital** |
| **F 3** | **Women referred from other health facility** |
| **F 4** | **Women referred to any higher level facility** |

**12. Please indicate whether any of the below mentioned interventions have been carried out (specify 0=no; 1=yes))**

| **Prevention of postpartum haemorrhage** | |  |  |  |
| --- | --- | --- | --- | --- |
| **G 0** | **Oxytocin** | **G 1** | **Other uterotonic** | |
| **Treatment of postpartum haemorrhage** | |  |  |  |
| **H 0** | **Oxytocin** | **H 5** | **Removal of retained products** | |
| **H 1** | **Other uterotonics** | **H 6** | **Balloon or condom tamponade** | |
| **H 2** | **Misoprostol** | **H 7** | **Artery litigation (uterin/hypogastric)** | |
|  |  | **H 8** | **Hysterectomy** | |
|  |  | **H 9** | **Abdominal packing** | |
| **Anticonvulsants** |  |  |  |  |
| **I 0** | **Magnesium sulfate** | **I 1** | **Other convulsant** | |
| **Antihypertensives** | |  |  |  |
| **I 2** | **Hydralazin** | **I 3** | **Aldomet** | |
| **Antibiotics** |  |  |  |  |
| **J 0** | **Prophylactic antibiotics during caesarean section** | |  |  |
| **J 1** | **Parental, therapeutic antibiotics** |  |  |  |
| **Fetal lung maturation** | |  |  |  |
| **K 0** | **Corticosteroids (betamethasone or dexamethasone)** | |  |  |
| **Please specify any underlying causes of deaths / Near Miss** | | | **please specify: 0= No; 1 = yes** | |
| **L 0** | **Pregnancy with abortive outcome (abortion/ectopic pregnancy)** | | | |
| **L 1** | **Obstetric haemorrhage** |  | **L 5 Medical/surgical/mental disease and complications** | |
| **L 2** | **Hypertensive disorders** |  | **L 6 Unanticipated complications of management** | |
| **L 3** | **Pregnancy-related infections** |  | **L 7** | **Coincidental condition** |
| **L 4** | **Other obstetric diseases/ complications** | | **L 8** | **Unknown** |
| **Please specify any other known contributory / associated conditions** | | | **please specify: 0= No; 1 = yes** | |
| **M 0** | **Anaemia** |  |  |  |
| **M 1** | **HIV infection** |  |  |  |
| **M 2** | **Previous caesarean section** |  |  |  |
| **M 3** | **Prolonged/obstructed labour** |  |  |  |
| **M 4** | **Other condition** | **please specify** | **_____________________________________** | |
| **M 5** | **Other condition** | **please specify** | **______________________________________** | |
